# Special Issue: Lipid Metabolism, Adipogenesis and Fat Tissue Metabolism: Gene Regulation

**DOI:** 10.3390/genes14051121

**Published:** 2023-05-22

**Authors:** Marek Skrzypski, Paweł A. Kołodziejski

**Affiliations:** Department of Animal Physiology, Biochemistry, and Biostructure, Poznan University of Life Sciences, 60-637 Poznan, Poland

Lipid metabolism is pivotal in controlling energy homeostasis. Impaired lipid metabolism is a hallmark of numerous health problems, including adiposity, cardiovascular diseases, diabetes, and several types of cancers [1]. This Special Issue, which encompasses six original works and two review articles, aimed to focus on recent advances regarding contributions related to gene-regulated mechanisms responsible for lipid metabolism regulation, adipogenesis, and biological functions of adipose tissue. 

Growing evidence demonstrates that the lipid metabolism of peripheral tissues is modulated by peptide hormones [2]. Leciejewska et al. focused on the role of the spexin hormone in controlling the development and metabolism of muscle cells [3]. The authors demonstrated that spexin promotes the replication of the skeletal muscle cell line C2C12 and the differentiation of these cells into muscles. Their results showed that spexin may be involved in controlling the metabolism and formation of muscles. Another peptide hormone, adropin, was studied by Jasaszwili et al., who demonstrated that it stimulates lipolysis in 3T3–L1 and rat primary adipocytes [4]. Furthermore, it was shown that adropin upregulates the expression of adiponectin while suppressing the expression of visfatin. This study indicated that a decline might contribute to the regulation of metabolism through direct interaction with mature white adipocytes.

There is growing evidence that the metabolism of lipids is influenced by genetic variability, different miRNAs, and epigenetic modifications [5,6]. Alanbaei et al. revealed an association between ANGPTL3 gene variants and levels of irisin and lipid metabolism and insensitivity to insulin in Arab individuals from Kuwait [7]. Płatek et al. attempted to identify epigenetic modulations related to high levels of FGF-21 in obese non-diabetic individuals [8]. This study found that obese individuals with increased levels of FGF21 are characterized by alerted methylation in several genes encoding for a protein involved in regulating FGF-21 expression, its receptors, and cofactors. Furthermore, this study identified several miRNAs that may contribute to regulating FGF-21 expression and production in obesity. Hicks et al. used combined miRNome and transcriptome data to provide detailed characterization of miRNA-regulatory networks implicated in the development and functions of adipose tissue in the chick peri-hatching period [9]. Meanwhile, in their study, Małodobra-Mazur et al. investigated the influence of different fatty acids on the adipogenesis of subcutaneous- and visceral-derived mesenchymal stem cells [10]. This study identified metabolic differences between subcutaneous and visceral fat depots and showed that oleic acid has the most notable effect on adipogenesis.

This Special Issue also includes two review articles. Our research group discussed the potential role of selected peptide hormones discovered in the present century (adropin, apelin, elabela, irisin, kisspeptin, MOTS-c, phoenixin, spexin, and neuropeptides B and W) in controlling the formation and biology of white and brown adipocytes [11]. Furthermore, Dixon et al. summarized and elaborated on recent evidence highlighting the importance of lipases and nuclear receptors, PPARs, and liver X receptor (LXR) in obesity, diabetes, and non-alcoholic fatty liver disease [12].

In conclusion, the experimental data published in this SI significantly improved our knowledge of the role of peptide hormones in controlling lipid metabolism in adipose tissue and other peripheral tissues such as muscles. This SI also sheds light on the importance of miRNAs expression and epigenetic mechanism in controlling lipid metabolism, adipose tissue development, and its endocrine and metabolic activity. We believe the published results will play a key role in future research aiming to identify new diagnostic and therapeutic tools in lipid metabolism abnormalities and adipose dysfunction.
